# Identifying Patients at Risk of Acute Kidney Injury Among Medicare Beneficiaries With Type 2 Diabetes Initiating SGLT2 Inhibitors: A Machine Learning Approach

**DOI:** 10.3389/fphar.2022.834743

**Published:** 2022-03-11

**Authors:** Lanting Yang, Nico Gabriel, Inmaculada Hernandez, Scott M. Vouri, Stephen E. Kimmel, Jiang Bian, Jingchuan Guo

**Affiliations:** ^1^ Department of Pharmacy and Therapeutics, University of Pittsburgh, Pittsburgh, PA, United States; ^2^ Division of Clinical Pharmacy, Skaggs School of Pharmacy and Pharmaceutical Sciences, University of California, San Diego, San Diego, CA, United States; ^3^ Department of Pharmaceutical Outcomes and Policy, University of Florida, Gainesville, FL, United States; ^4^ Department of Epidemiology, University of Florida, Gainesville, FL, United States; ^5^ Department of Health Outcomes and Biomedical Informatics, University of Florida, Gainesville, FL, United States

**Keywords:** AKI (acute kidney injury), SGLT2i, machine learning (ML), type 2 dabetes, medicare

## Abstract

**Introduction:** To predict acute kidney injury (AKI) risk in patients with type 2 diabetes (T2D) prescribed sodium-glucose cotransporter two inhibitors (SGLT2i).

**Methods:** Using a 5% random sample of Medicare claims data, we identified 17,694 patients who filled ≥1 prescriptions for canagliflozin, dapagliflozin and empagliflozin in 2013–2016. The cohort was split randomly and equally into training and testing sets. We measured 65 predictor candidates using claims data from the year prior to SGLT2i initiation. We then applied three machine learning models, including random forests (RF), elastic net and least absolute shrinkage and selection operator (LASSO) for risk prediction.

**Results:** The incidence rate of AKI was 1.1% over a median 1.5 year follow up. Among three machine learning methods, RF produced the best prediction (C-statistic = 0.72), followed by LASSO and elastic net (both C-statistics = 0.69). Among individuals classified in the top 10% of the RF risk score (i.e., high risk group), the actual incidence rate of AKI was as high as 3.7%. In the logistic regression model including 14 important risk factors selected by LASSO, use of loop diuretics [adjusted odds ratio (95% confidence interval): 3.72 (2.44–5.76)] had the strongest association with AKI incidence.

**Disscusion:** Our machine learning model efficiently identified patients at risk of AKI among Medicare beneficiaries with T2D undergoing SGLT2i treatment.

## Introduction

Type 2 diabetes (T2D) affects over 400 million people globally. (Chen et al., 2011). The management of T2D is complex and challenging since it involves the prevention of organ damage and complications such as cardiovascular and kidney events. (Nathan, 1993; Zheng et al., 2018). Sodium glucose cotransporter two inhibitors (SGLT2i) have shown promise in preventing cardiovascular disease (CVD) and renal function decline in patients with T2D (Birkeland et al., 2017; Zaccardi et al., 2016) Conversely, SGLT2i agents have been associated with increased risk of acute kidney injury (AKI), likely attributed to hypovolemia attributable to its diuretic effect. (Nadkarni et al., 2017). AKI is a concerning adverse effect because it can increase the risk of end-stage renal disease and death.

Previous safety studies have focused on examining the association between SGLT2i use and occurrence of AKI (Menne et al., 2019); however, little is known about which factors increase the risk of AKI while on SGLT2i therapy. Identifying patients at increased risk of AKI and using SGLT2i is necessary to individualize SGLT2i treatment and improve CVD and renal outcomes in T2D patients while minimizing risks. The development of a high-performance predictive model can aid clinicians in balancing risks vs. benefits when prescribing SGLT2is. To address this evidence gap, we developed a machine learning model to predict the risk of AKI among T2D patients undergoing SGLT2i treatment, and identified risk factors associated with incident AKI after SGLT2i initiation.

## Materials and Methods

### Study Population and Follow-Up

The study used 2012–2016 claims data from a 5% random sample of Fee-for-Service Medicare beneficiaries. We first identified patients with T2D and those with at least one SGLT2i prescription (canagliflozin, dapagliflozin, or empagliflozin) filled between April 2013 and December 2016. T2D diagnosis was defined following the Center for Medicare and Medicaid Services (CMS) Chronic Condition Warehouse (CCW) definition, which traces back the first diagnosis to the first month of Medicare eligibility. (Service CfMaM (2020). Chr, 2020). The date of the first SGLT2i prescription filled during the study period was designated as index date. We excluded patients who did not have continuous Medicare Part D enrollment in the year prior to index date (i.e., baseline year) or who had filled a prescription for SGLT2i in the baseline year.

Patients were followed from index date until AKI incidence, therapy discontinuation (defined as a treatment gap ≥60 days), death, or the end of the study (31 December 2016). This study was approved by the institutional review board at the University of Pittsburgh as an exempt study because de-identified data were used in analyses.

### Outcome

The outcome variable was an incident AKI event after index date, which was defined as having inpatient primary or secondary diagnosis of International Classification of Disease (ICD), Ninth Revision diagnosis codes 584, or 10th Revision primary diagnosis code N17. (D'Arienzo et al., 2019).

### Predictors

We compiled 65 predictor candidates using claims data from the baseline year, including information on sociodemographics, diabetes duration, comorbidities, and other medications (Supplemental Table S1). Sociodemographic characteristics included age, sex, race, region of residence, Medicaid eligibility, and receipt of low-income subsidy. Comorbidity information included history of AKI, acquired hypothyroidism, Alzheimer disease, ischemic heart disease, stroke or transient ischemic attack, atrial fibrillation, anemia, congestive heart failure, hyperlipidemia, hypertension, chronic kidney disease, chronic obstructive pulmonary disease, lower extremity amputations, peripheral vascular disease, prostatic hyperplasia, rheumatoid arthritis, breast cancer, lung cancer and prostate cancer. Medication use included the use of other antidiabetic classes (i.e., metformin, sulfonylureas, thiazolidinediones, dipeptidyl peptidase four inhibitors, glucagon-like peptide-1agonists, insulin, and others), angiotensin converting enzyme inhibitors (ACEi), angiotensin receptor blockers (ARB), nonsteroidal anti-inflammatory drugs (NSAIDS), antiplatelet and loop diuretics. Predictors candidates were selected based on the risk factors associated with AKI in previous studies. (Nadkarni et al., 2017; Bhatraju et al., 2019; Kalisvaart et al., 2019; Menne et al., 2019).

### Statistical Analyses

We split the sample randomly and equally into training (*n* = 8,847) and testing (*n* = 8,847) sets. We employed three machine learning approaches, including least absolute shrinkage and selection operator (LASSO), elastic net, and random forest (RF) to develop the AKI prediction model (Supplemental Table S2), which have been commonly used and shown success in predicting health outcomes. We used the training set to develop machine learning models and perform hyperparameter tuning and the testing set to evaluate the prediction performance.

C-statistics (or area under the receiver operating characteristic curves) including 95% confidence interval (CI) that were estimated by bootstrapping were calculated to assess the model’s discrimination performance in the testing set. We plotted the observed AKI incidence rate across different risk subgroups (i.e., ≤50th, 50–75th, 75th–90th, and >90th percentile of the machine learning risk score) in the testing set to evaluate calibration performance.

To obtain the unbiased estimation of risk factors’ odds ratios, we constructed a logistic regression model by including risk factors selected by LASSO (Supplemental Table S3). Analyses were performed using SAS, Version 9.4 (SAS Institute Inc.), and Python, Version 3.7 (Python Software Foundation).

## Results

Among 17,694 beneficiaries who initiated on SGLT2i, 194 (1.10%) developed AKI over a median follow-up period of 1.5 years. Patients in the training and testing sets shared comparable distributions of characteristics; both samples were approximately 48% male and 75% White (Supplemental Table S4).

**FIGURE 1:**
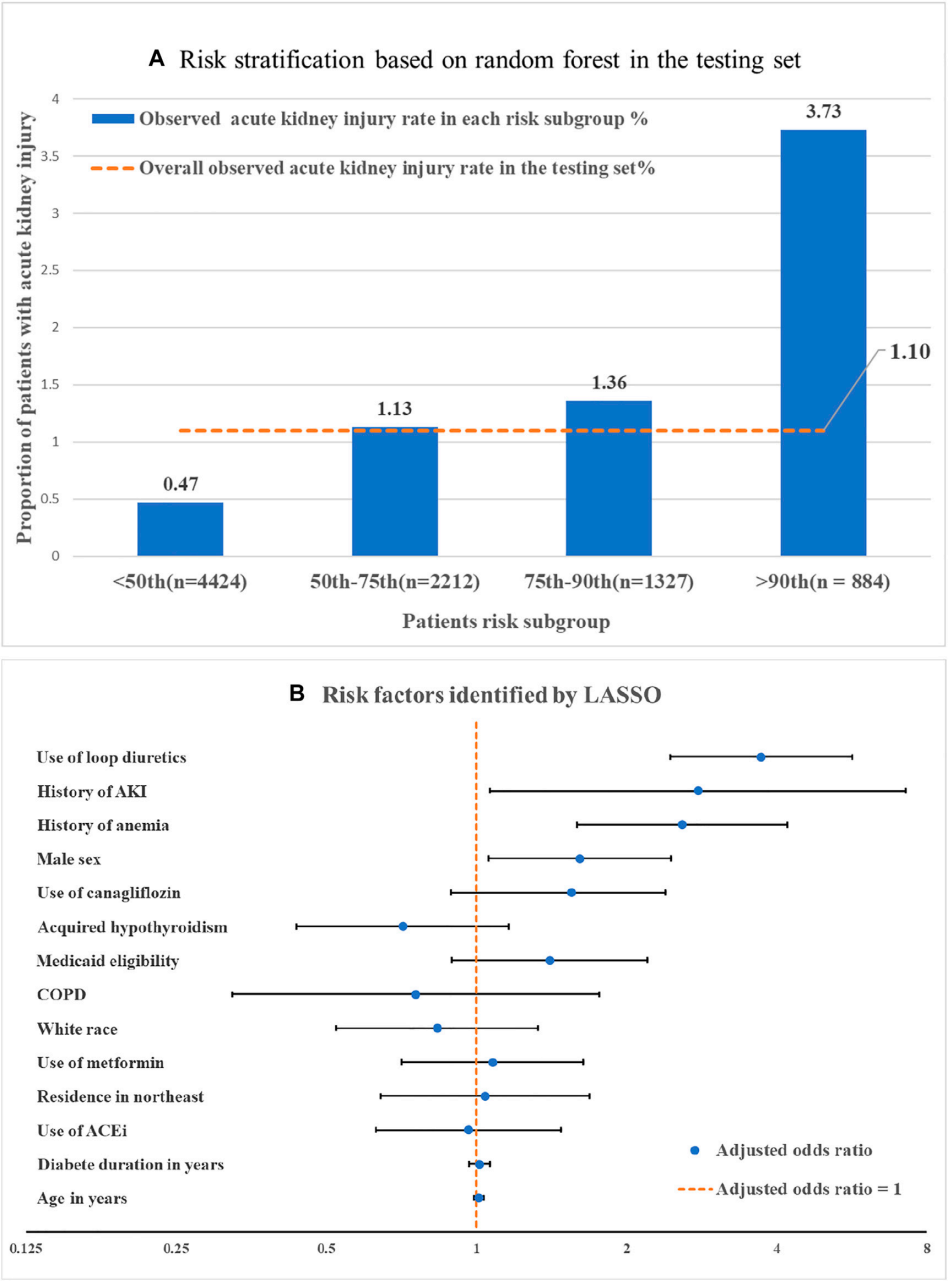
Machine learning based risk stratification and important features. **(A)** Risk stratification by machine learning generated risk score: risk subgroup was categorized by the risk score that generated by random forest (i.e., ≤50th, 50–75th, 75–90th, >90th percentile of the random forest risk score). **(B)** Risk factors identified by LASSO. Adjusted odds ratios were obtained by regressing LASSO selected features against the AKI outcome a multiple logistic regression model. Note: The comparator for white race was non-white race, for residence in northwest was non residence in northwest. The comparator for use of canagliflozin was use of other SGLT2i including dapagliflozin and empagliflozin. Abbreviations: LASSO, Least absolute shrinkage and selection operator-type regularized regression. AKI, Acute kidney injury; COPD, chronic obstructive pulmonary disease; ACEi, Angiotensin-converting enzyme inhibitors.

The RF (C-statistic = 0.72, 95% CI 0.68–0.76) outperformed LASSO (C-statistic = 0.69, 95% CI 0.65–0.73) and elastic net (C-statistic = 0.69, 95% CI 0.65–0.73) in predicting AKI among T2D patients initiating SGLT2i. Using the risk score generated by the RF model, we categorized patients into four risk subgroups (Figure 1A). In the highest risk (i.e., top 10% of the risk score) and lowest risk (i.e., bottom 50% of the risk score) groups, the observed AKI incidence rates were 3.73% and 0.47%, respectively.

We identified risk factors using LASSO because of the interpretability of predictors in linear-based algorithms. Among 14 important features selected by LASSO (Figure 1B), use of loop diuretics [adjusted odds ratio (aOR): 3.72, 95% CI 2.45–5.6] and history of AKI (aOR: 2.78, 95% CI 1.06–7.3) had the strongest association with AKI incidence (Figure 1B), followed by anemia (aOR: 2.56, 95% CI 1.59–4.20) and male sex (aOR: 1.61, 95% CI 1.06–2.47).

## Discussion

Using real-world data, this study successfully developed and validated a machine learning model to identify T2D patients who were at high risk of AKI after SGLT2i initiation. The tree-based machine learning algorithm, RF, had reasonably good predictive utility and efficiently classified individuals into different risk subgroups. Although the predictive performance of LASSO––the linear-based algorithm––was slightly inferior to RF, it identified important risk factors associated with the development of AKI. The most important predictor was the use of loop diuretics which was associated with an almost four-fold increase in the odds of developing AKI among T2D patients taking SGLT2i.

To the best of our knowledge, our study was the first to identify an increased risk from loop diuretics among SGLT2i users. Traditionally, loop diuretics had been used to relieve symptoms of heart failure. The concomitant use of SGLT2i and loop diuretics may become common, given the remarkable reduction effect from SGLT2i in HF-associated negative outcomes. (Zaccardi et al., 2016; Wilcox et al., 2018). However, the synergistic effect of loop diuretics and SGLT2i in volume depletion might result in hypovolemia and systemic hypoperfusion, and further lead to reduced renal blood flow and AKI. (Wilcox et al., 2018; Mordi et al., 2020; Goyal et al., 2021). Future studies are needed to examine the safety profile of the combined use of loop diuretics and SGLT2 inhibitors among real-world patients.

To the best of our knowledge, our study is the first to use machine learning methods to predict AKI risk among real-world patients treated with SGLT2i. Previous AKI prediction models mainly focused on patients in critical and perioperative care. (Adhikari et al., 2019; Bhatraju et al., 2019; Kalisvaart et al., 2019; Gameiro et al., 2020). Our examination of patients is SGLT2is of major relevance due to the exponential uptake of SGLT2is in the recent years, driven by their distinctive cardiorenal benefits. (Zaccardi et al., 2016; Birkeland et al., 2017). Employing machine learning algorithms and administrative health care data to predict such rare, but serious, adverse events can provide opportunities for optimizing clinical decision making and individualized patient care. A risk prediction tool might be developed and implemented at the point of care to assist with therapeutic decisions and reduce preventable drug-related adverse outcomes. Nevertheless, future studies are needed to improve the performance of the model. Using claims data, we were not able to incorporate important predictors for SGLT2i users in the model such as serum creatinine level, hemoglobin A1c level. Our model performance can be further enhanced by using other more robust databases such as electronic health records databases. Furthermore, our model need to be updated for data that are more current considering the up taking trend of SGLT2i use. Lags in claim data is a hurdle that must be addressed prior to the implementation of the model.

The current study is subject to limitations. First, using claims data, we were unable to include some of the important clinical information used to predict AKI outcomes, such as estimated glomerular filtration rate (eGFR) and blood pressure. Second, our model was developed among new users of SGLT2i and predicted AKI risk within 1.5 years of follow-up. Future studies are needed to update the current model by using advanced methods and more robust linked data to dynamically predict AKI over the course of SGLT2i treatment. Third, external validation is needed for the current prediction model. Fourth; our findings may not be generalizable to other populations and settings, considering that the study was conducted among older adults with T2D based on Fee-for-Service Medicare claims data.

## Conclusion

We successfully developed a machine learning model to predict AKI risk among T2D patients under SGLT2i treatment. Important risk factors––including use of loop diuretics, a history of AKI, and anemia––were identified as being associated with AKI development. Our data revealed relevant unmet needs that future studies can address through the implementation of machine learning-based alert tools aimed to improve early identification of rare, but serious, drug-related adverse events to support clinical, therapeutic decision-making and maximize treatment benefits.

## Data Availability

The data analyzed in this study was obtained from Center for Medicare and Medicaid Services, the following licenses/restrictions apply: the data is not publicly available due to user agreement restrictions. Requests to access these datasets should be directed to Jingchuan Guo, juoj1@cop.ufl.edu.
