# Supplementary material for: Identifying Patients at Risk of Acute Kidney Injury Among Medicare Beneficiaries With Type 2 Diabetes Initiating SGLT2 Inhibitors: A Machine Learning Approach
Source: Front Pharmacol. 2022 Mar 11;13:834743. doi: 10.3389/fphar.2022.834743 (PMC8961669; doi:10.3389/fphar.2022.834743)
Supplement: Supplementary file 1 [file DataSheet1.docx]

Supplemental materials

Supplemental Table 1 Predictors candidates including in machine learning models

| **Sociodemographic** | **History of disease** | | **Medication use** |
| --- | --- | --- | --- |
| Age (years) | Acquired hypothyroidism | End stage renal disease | ACEi |
| Sex | Acute kidney injury | Endometrial cancer | Antiplatelet |
| Race | Alzheimer disease | Glaucoma | ARBs |
| White | Alzheimer's disease related disorders, or senile dementia | Hypertension | BAS |
| Black | Acute Myocardial Infarction | Hyperlipidemia | DPP-4 inhibitors |
| Hispanic | Anemia | Ischemic heart disease | GLP-1 agonists |
| Asian | Asthma | Liver disease | Insulin |
| Medicaid eligibility | Atrial Fibrillation | Lower extremity amputation | Loop diuretics |
| Receipt of low-income subsidy | Breast cancer | Lung cancer | Meglitinides |
| Diabetes durations(years) | Cataract | Mortality | Metformin |
| Residence region | Chronic kidney disease | Peripheral vascular disease | NSAIDs |
| Midwest | Colorectal cancer | Prostate cancer | Sulfonylureas |
| Northeast | Congestive heart failure | Prostatic hyperplasia | TZD |
| Southeast | Chronic obstructive pulmonary disease | Rheumatoid arthritis / osteoarthritis | Canagliflozin |
| Southwest | Depression | Stroke or TIA | Dapagliflozin |
| West |  |  | Empagliflozin |

Abbreviations: TIA = Transient ischemic stroke; ACEi = Angiotensin converting enzyme inhibitors; ARBs =Angiotensin receptor blockers; BAS = Bile acid sequestrants; DPP-4 =Dipeptidyl peptidase 4; GLP-1 = Glucagon-like peptide-1; NSAIDS = Nonsteroidal anti-inflammatory drugs; TZD = Thiazolidinediones

| Name | Type of Machine learning algorithms | Advantages | Limitations |
| --- | --- | --- | --- |
| LASSO | Linear-based | - Not only punishing high values of the coefficients but setting the value to zero if the variable is not relevant; - Performing automatic feature selection; - Providing interpretable coefficients; | - Feature selection with bias; - LASSO will select only one feature from a group of correlated features; |
| Elastic net | Linear-based | - Increasing the flexibility and stability of feature selection by penalizing both absolute and square size; - Could deal with the situations where the number of features is over the number of samples; | - Computationally expensive; - Double shrinkage introduce more bias compare to LASSO; |
| Random forest | Ensemble-based | - Lower chance of variance and overfitting of training data compared to decision trees; - Scales well for large datasets; - Offering estimates of variable importance; | - RF favors those variables or attributes that can take high number of different values in estimating variable importance; - Less interpret-ability; |

Supplemental Table 2 Comparison of three algorithms

| Statistical analyses | Software | Sample codes |
| --- | --- | --- |
| Random forest | Python, Version 3.7 | from sklearn.ensemble import RandomForestClassifier  rf = RandomForestClassifier(bootstrap =False,  max_depth = 2, max_features ='log2',  min_samples_leaf=1000, min_samples_split= 2,  min_weight_fraction_leaf= 0, n_estimators =500,  class_weight={0:1, 1:1},  oob_score =False, random_state = 0)  rf.fit(x,y) |
| Least absolute shrinkage and selection operator (LASSO) | Python,  Version 3.7 | from sklearn.linear_model import Lasso  lasso = Lasso(alpha=0.01, copy_X=True, fit_intercept=True, max_iter=50,  normalize=False, positive=False, precompute=False, random_state=0,  selection='cyclic', tol=1, warm_start=False)  lasso.fit(x,y) |
| Elastic net | Python,  Version 3.7 | from sklearn.linear_model import ElasticNet  enet = ElasticNet(alpha=0.01, copy_X=True, fit_intercept=True,l1_ratio=0.05,  max_iter=500,normalize=False, positive=False, precompute=False,  random_state=0, selection='cyclic', warm_start=False)  enet.fit(x,y) |
| Logistic regression | SAS, Version 9.4 | proc logistic data=x ;  class race treatment(ref= “canagliflozin”);  model aki = treatment age sex race aki_history;  run; |

Supplemental Table 3 Sample codes for machine learning algorithms and the logistic regression model

Supplemental Table 4 Baseline patient characteristic

| **Patients characteristics** | **Training set** | **Testing set** |
| --- | --- | --- |
|  | **(N=8,847)** | **(N=8,847)** |
| **Sociodemographic** |  |  |
| Age in years | 67.4(10.5) | 67.2(10.4) |
| Diabetes Duration | 6.5(4.6) | 6.4(4.6) |
| Male | 4286(48.5%) | 4249(48.0%) |
| Race |  |  |
| White | 6720(76.0%) | 6639(75.0%) |
| Black | 1096(12.4%) | 1079(12.2%) |
| Hispanic | 418(4.7%) | 445(5.0%) |
| Asian | 314(3.6%) | 331(3.7%) |
| Residence Region |  |  |
| Middlewest | 1701(19.2%) | 1645(19.0%) |
| Northeast | 1918(21.7%) | 1965(22.2%) |
| Southeast | 2860(32.3%) | 2828(32.0%) |
| Southwest | 1032(11.7%) | 1064(12.0%) |
| Westeast | 1292(14.6%) | 1297(14.7%) |
| Receipt of low-income subsidy | 5583(63.1%) | 5606(63.4%) |
| Medicaid eligibility | 3526(39.9%) | 3507(39.6%) |
| **Clinical characteristics** |  |  |
| Incidence of acute kidney injury | 97(1.1%) | 97(1.1%) |
| History of acute kidney injury | 96(1.1%) | 100(1.1%) |
| Anemia | 4129(46.7%) | 4126(46.6%) |
| Asthma | 1538(17.4%) | 1577(17.8%) |
| Atrial Fibrillation | 169(1.9%) | 148(1.7%) |
| Chronic kidney disease | 251(2.8%) | 230(2.6%) |
| Congestive heart failure | 480(5.4%) | 465(5.3%) |
| COPD | 522(5.9%) | 461(5.2%) |
| Hyperlipidemia | 7815(88.3%) | 7739(87.5%) |
| Hypertension | 8017(90.6%) | 7896(89.3%) |
| Ischemic heart disease | 1587(17.9%) | 1534(17.3%) |
| Stroke or TIA | 168(1.9%) | 140(1.6%) |
| Use of ACEi | 3090(35.0%) | 3009(34.0%) |
| Use of ARBs | 2601(29.4%) | 2676(30.2%) |
| Use of loop diuretics | 1340(15.2%) | 1321(15.0%) |
| Use of NSAIDS | 1381(15.6%) | 1337(15.1%) |
| Use of metformin | 5443(61.5%) | 5512(62.3%) |
| Use of DPP-4 inhibitors | 2796(31.6%) | 2861(32.3%) |
| Use of insulin | 2693(30.4%) | 2760(31.2%) |
| Use of sulfonylureas | 3375(38.2%) | 3365(38.0%) |
| Use of thiazolidinediones | 745(8.4%) | 674(7.6%) |
| Use of GLP-1 agonists | 10545(11.9%) | 1021(11.5%) |
| Use of meglitinides | 147(1.7%) | 166(1.9%) |

Abbreviations: COPD = Chronic obstructive pulmonary disease; TIA = Transient ischemic stroke; ACEi = Angiotensin converting enzyme inhibitors; ARBs =Angiotensin receptor blockers; GLP-1 = Glucagon-like peptide-1; NSAIDS = Nonsteroidal anti-inflammatory drugs; DPP-4 =Dipeptidyl peptidase 4 ;

Continuous variables are expressed as mean and standard deviation (square baskets). Categorical variables are expressed as frequency and percentages (square brackets).
